# Ensuring safety and efficacy in combination products: regulatory challenges and best practices

**DOI:** 10.3389/fmedt.2024.1377443

**Published:** 2024-07-10

**Authors:** Deepak Kumar Gupta, Akhilesh Tiwari, Yashraj Yadav, Pranay Soni, Megha Joshi

**Affiliations:** ^1^ Department of Pharmacy, Indira Gandhi National Tribal University, Amarkantak, India; ^2^ Department of Pharmacology, Acropolis Institute of Pharmaceutical Education and Research, Indore, India; ^3^ Institute of Pharmacy, Vikram University, Ujjain, India

**Keywords:** combination product, regulatory challenges, pharmacovigilance, case study, best practices

## Abstract

Combination products, amalgamating drugs, biologics, and medical devices, have revolutionized the healthcare landscape with their potential for innovative therapies. However, the intersection of diverse components within these products presents a complex regulatory environment, demanding rigorous attention to safety and efficacy. This article delves into the intricate landscape of regulatory considerations, safety, and efficacy assessments pertaining to combination products—a category at the intersection of drugs, devices, and biologics. The regulatory framework, primarily governed by the U.S. Food and Drug Administration (FDA), necessitates a nuanced classification determining the regulatory pathway. Collaboration between diverse regulatory centers, such as the Center for Drug Evaluation and Research (CDER) and the Center for Devices and Radiological Health (CDRH), underscores the integrated approach required for these innovative healthcare solutions. Safety considerations unravel the potential risks and adverse events associated with combining diverse components, emphasizing the need for robust risk assessment and mitigation strategies. The evaluation of efficacy involves sophisticated methodologies, clinical trials, and post-market surveillance, with recent advancements incorporating digital technologies. This comprehensive exploration aims to contribute to the evolving understanding and best practices in the regulatory and scientific realms, fostering collaboration and innovation in the development and assessment of combination products.

## 1 Introduction

In the realm of modern healthcare, the convergence of pharmaceuticals, medical devices, and biologics has given birth to a remarkable class of medical interventions known as combination products (1). Combination products have the potential to give greater therapeutic benefits than single-entity devices such as medicines and biologics (2). Combination products are therapeutic and diagnostic things that contain a combination of medications, devices, and/or biological elements (3).

These innovative and multifaceted healthcare solutions have reshaped the landscape of patient care, offering novel treatment modalities that hold great promise (4). From drug-eluting stents to combination vaccines, combination products have demonstrated their potential to enhance therapeutic outcomes, improve patient adherence, and address some of the most challenging healthcare issues (5).

**Figure 1:**
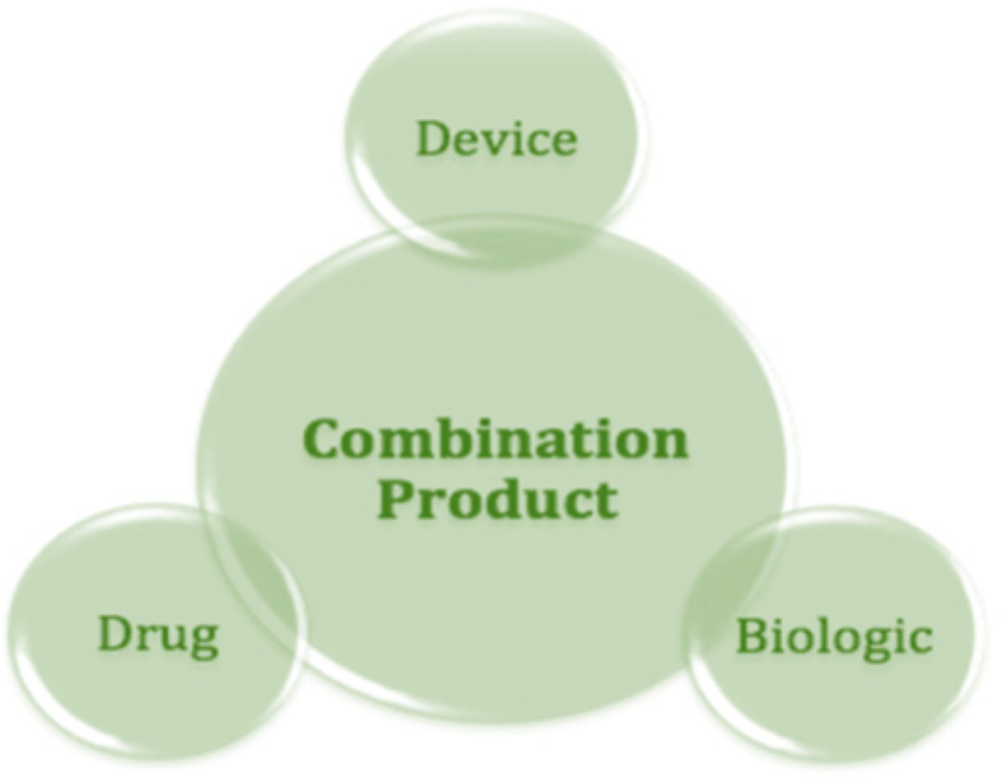
Development of combination product.

Figure 1 is visually represents the intricate process of developing combination products, which integrate drugs, devices, and biologics to create innovative healthcare solutions. Figure likely outlines the various stages of development, from conceptualization to market launch, illustrating key steps such as research, design, regulatory approval, and manufacturing. It serves as a visual guide, highlighting the collaborative efforts required among interdisciplinary teams to navigate the complexities of combination product development effectively.

The significance of combination products in healthcare lies not only in their ability to deliver integrated and synergistic treatments but also in their potential to address unmet medical needs (6). By bringing together different therapeutic approaches, they offer unique advantages that can translate into improved patient quality of life and better clinical outcomes (7). The ever-increasing pace of innovation in this field underscores their importance as a driving force in the ongoing advancement of medical science (8).

However, this promise comes with a critical caveat: the need for stringent regulatory oversight. The amalgamation of drugs, devices, and biologics in combination products creates a complex and multifaceted landscape where safety and efficacy are paramount (9). Ensuring that these products deliver their intended benefits without causing harm requires a meticulous and multifaceted approach to regulatory control (10).

Regulatory authorities, such as the U.S. Food and Drug Administration (FDA) and their counterparts worldwide, play a vital role in safeguarding public health (11). They navigate complex and evolving landscapes to establish regulatory frameworks that govern combination products, a class of medical interventions that do not fit neatly into traditional categories (12). The importance of this oversight cannot be understated, as it is inextricably linked to patient safety and the efficacy of these innovative medical solutions (13).

## 2 Regulatory challenges for combination products

Regulatory Operations' Significant Obstacles. Some of the factors to consider when navigating the regulatory operations for drug-device combos are like: Keeping up-to-date with recent regulatory standards and revisions, recognizing product categorization and regulatory pathways, Taking care of the scientific and technical requirements, Patient care as well as usability testing, Considerations after the market (14, 15). Regulatory challenges in the realm of combination products arise from their unique nature, blending drugs, devices, and biologics, and require manufacturers and regulatory agencies to address several key complexities (16). These challenges include the intricate process of classifying these products, determining their primary mode of action (PMOA), and choosing the appropriate regulatory pathway (17). Overlapping regulations further complicate matters, with some combination products falling under the purview of multiple regulatory authorities (18). For instance, devices combined with biologics may be subject to both device and biologics regulations. The rapid evolution of healthcare technology adds to the challenge, with the integration of digital technologies in combination products demanding adaptive regulatory guidelines (19). post-market surveillance for ongoing safety and efficacy, along with the need to harmonize international regulations for global market entry, introduce additional complexities. To illustrate, consider the example of drug-eluting stents, where classifying these combination products requires a nuanced assessment of the mechanical action of the stent and the pharmacological action of the drug component (20). Furthermore, wearable combination products like insulin pumps challenge traditional regulatory boundaries, necessitating updated guidelines to accommodate technological innovations (21). Combination vaccines require comprehensive post-market surveillance, given the complexity of managing different components' adverse events and interactions (22). To overcome these regulatory challenges, collaboration between manufacturers and regulatory authorities, interdisciplinary expertise, and staying informed about evolving regulations are essential to ensure the safety and efficacy of combination products (23).

**Figure 2:**
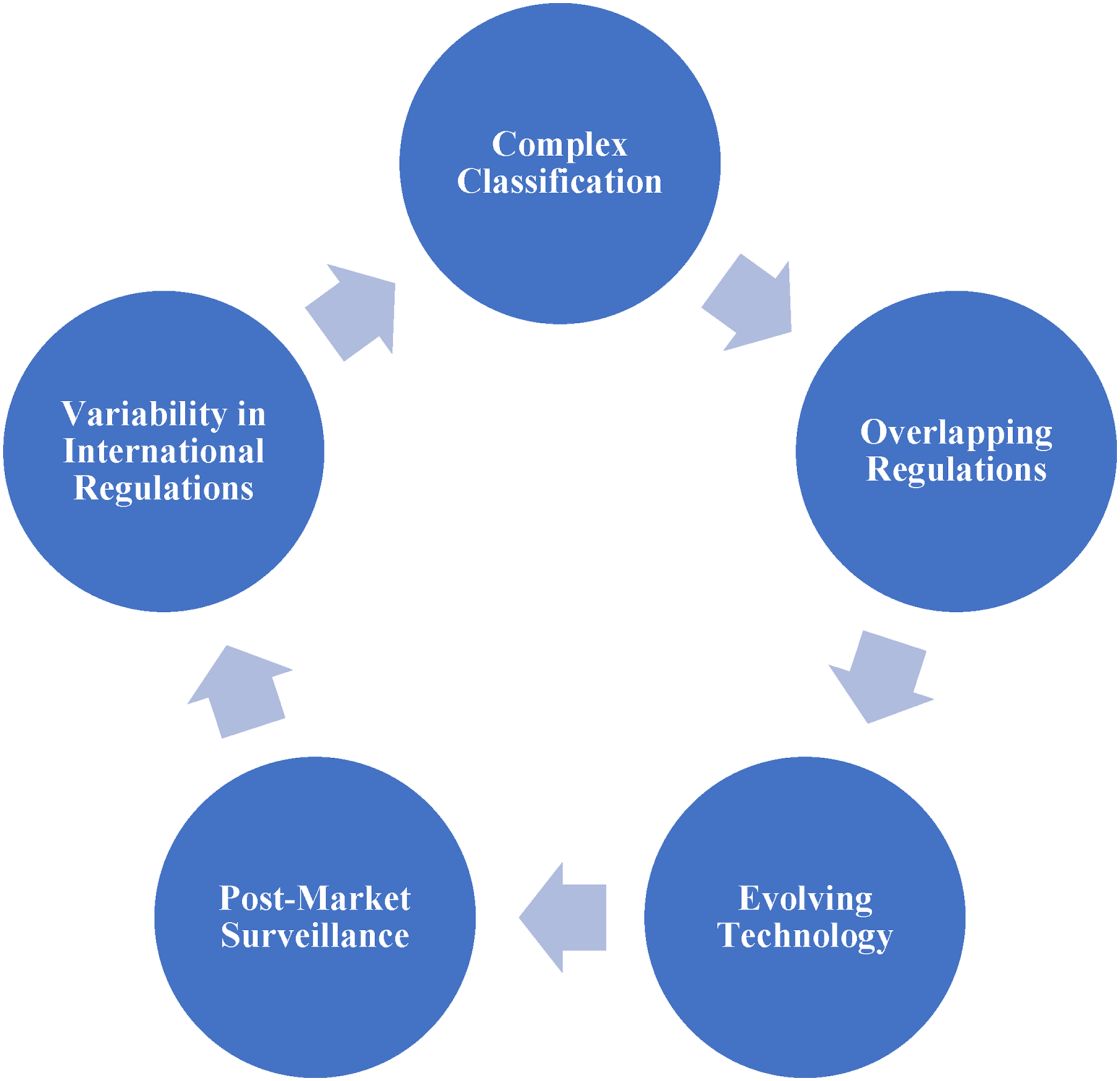
Regulatory challenges.

Figure 2 provides readers with a visual representation of the multifaceted regulatory challenges inherent in the development and approval of combination products, emphasizing the importance of comprehensive regulatory strategies and collaboration in addressing these challenges effectively.

### 2.1 Case studies

#### 2.1.1 Drug-eluting stents

Drug-eluting stents' classification is complex due to the need to consider both the mechanical action of the stent and the pharmacological action of the drug component (20).

#### 2.1.2 Wearable combination products

Wearable's integrated with pharmaceuticals, like insulin pumps, challenge traditional regulatory boundaries, requiring adaptive guidelines (24).

#### 2.1.3 Combination vaccines

Combination vaccines demand long-term post-market surveillance to address varying components' adverse events and interactions (25).

Table 1 a clear and organized overview of various combination products and their regulatory classifications, aiding in the understanding of the regulatory landscape for these innovative healthcare interventions.

**Table 1:** Different types of combination products, their components, regulatory pathways, and examples.

Combination Product	Components	Regulatory Pathway	Examples
Drug-eluting Stents	Drug, Medical Device	Device or Biologics	Xience V, Promus Premier, Resolute Integrity
Wearable Insulin Pump	Drug, Medical Device	Device or Biologics	Medtronic MiniMed 670G, Tandem t: slim X2
Combination Vaccines	Multiple Vaccines	Biologics	MMRV (Measles, Mumps, Rubella, Varicella)
Drug-Device Inhalers	Drug, Medical Device	Device or Biologics	Advair Diskus, Symbicort
Drug-Coated Balloons	Drug, Medical Device	Device	IN. PACT Admiral, Ranger

### 2.2 Challenges facing by regulatory agencies

Regulatory agencies are confronted with a multitude of challenges as they endeavor to oversee and regulate healthcare interventions and products, including the increasingly rapid pace of technological advancements that continually introduce novel and intricate medical solutions (26). The interdisciplinary nature of combination products, which combine drugs, devices, and biologics, poses complexities that demand extensive expertise and coordination. Resource constraints, encompassing both financial and human resources, often limit the capacity of these agencies. Moreover, the imperative of global harmonization necessitates the alignment of regulations and standards across countries (27). The changing risk profiles of emerging technologies and treatments demand that regulatory authorities develop the ability to effectively access and manage these risks, while post-market surveillance systems must be established to monitor safety and efficacy continually (28). Collaboration with other agencies, adaptation to evolving regulations, and maintaining transparency and open communication are pivotal. Furthermore, as public expectations and demands for transparency and responsiveness rise, regulatory agencies must navigate increased scrutiny (29). Additionally, bioethical challenges, such as those related to genetic editing or stem cell therapies, further complicate their tasks (30). In response to these multifaceted challenges, regulatory agencies are compelled to refine their approaches, update guidelines, and engage with various stakeholders to adapt to the changing landscape and to ensure the safety and efficacy of healthcare interventions (31).

### 2.3 New guidelines related regulatory agencies

EU to reform rules for drug-device combinations, product information and pharmacovigilance:

The European Union's new strategy for Europe, which set goals for a patient-centered environment where the EU industry may continue to develop and be a worldwide leader in the sector, came three years before the proposed legislative reform of the pharmaceutical market (32).

Key regulatory instruments, such as Directive 2001/83/EC, Regulation (EC) 726/2004, and the existing guidelines on pharmaceuticals for pediatric use and orphan medications, would be impacted by the reform plan unveiled in April 2023 (33).

By simplifying and updating the pharmaceutical regulatory system to reflect advancements in science, technology, and the environment, the reform seeks to guarantee that patients throughout the European Union have access to medications and that supplies are secure (34).

#### 2.3.1 For medical devices and combinations

Taking a cue from the US, the draught reform suggestions expand on the European Medicines Agency's (EU) 2017/745 Medical Device Regulation (MDR) guidelines to provide the first official EU definitions of drug-device combinations (35).

#### 2.3.2 For pharmacovigilance

Benefiting from the pharmacovigilance guidelines established by Directive 2001/83/EC, the proposal seeks to improve openness and communication among interested parties, including the public. Among the measures are, for example, a web portal connecting each Member State to the EMA's web portal, which serves as a communication tool for summaries of public assessment reports, product characteristics and package leaflets, risk management plans, and information on reporting suspected adverse product reactions to the Member State's competent authorities for medicinal products covered by a national MA (36).

## 3 Regulatory framework for combination product

The regulatory framework for combination products involves a meticulous process governed by the U.S. Food and Drug Administration (FDA) and other relevant regulatory authorities globally (37). In the United States, the FDA plays a central role, with the Office of Combination Products determining the primary mode of action to assign the appropriate regulatory pathway (38). This classification influences whether the product follows drug, device, or biologic regulations. The FDA collaborates with multiple centers, including the Center for Drug Evaluation and Research (CDER) and the Center for Devices and Radiological Health (CDRH), emphasizing an integrated approach to address safety and efficacy across diverse product components (39). The classification further guides the approval processes, with drug-dominated products following New Drug Application (NDA) or Biologics License Application (BLA) pathways, device-dominated products undergoing Premarket Approval (PMA) or 510(k) processes, and balanced products navigating a customized pathway based on primary mode of action (40). This intricate regulatory framework underscores the importance of collaboration, transparency, and adherence to evolving guidelines to ensure the effective development, evaluation, and approval of combination products that meet rigorous safety and efficacy standards (41).

Table 2 A comprehensive comparison of combination product regulations, aiding in navigating the regulatory landscape in different jurisdictions.

**Table 2:** Comparative regulatory framework for combination products.

Regulatory Aspect	United States	European Union	India
Governing Legislation	FDA Regulations	Medical Device Regulation (MDR), Pharmaceuticals Legislation	Drugs and Cosmetics Act of 1940, Drugs and Cosmetics Rules of 1945
Regulatory Authority	U.S. Food and Drug Administration (FDA)	European Medicines Agency (EMA), European Commission	Drug Controller General of India (DCGI), Central Drugs Standard Control Organization (CDSCO)
Product Classification	Office of Combination Products	Explicit definitions in MDR for drug-device combinations	Combination products categorized as Fixed Dose Combinations (FDCs)
Collaboration Between Authorities	Collaboration between FDA, CDER, and CDRH	Harmonized regulations, collaboration between EU Member States and EMA	Regulatory oversight by CDSCO under the Ministry of Health and Family Welfare
Post-Market Surveillance	Stringent requirements, continuous monitoring	Enhanced pharmacovigilance guidelines, centralized database	Manufacturers prove effectiveness and safety, multi-factorial research often required
Unique Regulatory Challenges	Nuanced assessment of primary mode of action (PMOA)	Evolving guidelines and definitions for drug-device combinations	Stricter requirements for proving functionality duration of active ingredients
ISO 13485 Certification Requirement	Applies to medical devices, not specifically combination products	Applies to medical devices, including drug-device combinations	Necessary for validating quality systems of production facilities
Notified Body Involvement (EU)	Not applicable	Involvement for CE mark approval, conformity assessments	Not applicable in the same context

## 4 Safety considerations for combination products

Safety considerations for combination products are of paramount importance, given their intricate fusion of drugs, devices, and biologics. These products introduce unique challenges as they may present unforeseen interactions and synergies. Potential risks and adverse events must be thoroughly explored, encompassing issues related to compatibility, potential device malfunctions, and the specific pharmacological effects of the incorporated drugs or biologics (42). Comprehensive risk assessment is crucial throughout the product life cycle, from development to post-market surveillance. Manufacturers must employ rigorous strategies to mitigate identified risks, ensuring user safety and product effectiveness (43). The importance of clear communication and education for healthcare professionals and patients cannot be overstated, contributing to informed use and proper risk management (44). Regulatory agencies play a vital role in overseeing these considerations, emphasizing a proactive and collaborative approach among manufacturers, healthcare providers, and regulatory bodies to ensure the highest standards of safety for combination products (45).

The Adverse Event (AE): Key Definitions (Source: ICH E2A) -Any adverse medical event that occurs in a patient or clinical research participant who is given a pharmaceutical product; the event need not be directly related to the treatment and same time (21 CFR 820.3) complaint -Any written, electronic, or spoken communication that makes assertions regarding the identity, composition, reliability, robustness, safety, effectiveness, or usage of a device after it is released for public use (46).

## 5 Efficacy assessment of combination products

Evaluating the efficacy of combination products involves a multifaceted approach that considers the integrated performance of drugs, devices, and biologics. Clinical trials form a cornerstone, employing rigorous methodologies and diverse endpoints to comprehensively assess the product's effectiveness (47). The selection of appropriate endpoints depends on the primary mode of action; with clinical, physiological, or patient-reported outcomes being common measures (48). Post-market surveillance is essential for continuous efficacy assessment, monitoring real-world performance and potential long-term effects. Recent advancements emphasize the integration of digital technologies, such as real-world evidence and wearable devices, offering dynamic insights beyond traditional trial settings (49). Best practices include the establishment of robust post-market surveillance systems, close collaboration between manufacturers and regulatory authorities, and ongoing adaptation to emerging methodologies, ensuring a thorough and contemporary evaluation of the efficacy of combination products (50).

Drug Container Closure and Device Constituent Part Evaluations Are Included in Biological Safety Assessments for Drug-Device Combination Products. Both device and drug-based packaging standards have been deemed applicable when drug delivery systems and drug container closure systems are included in the device's constituent parts (51).

In contrast to pharmaceutical package delivery systems, other methods are employed for the bioassessment of medical equipment. Among the variations is the application of toxicological evaluation and chemical characterization either in place of or in addition to biological *in vivo*/*in vitro* testing (52). There are variations in how nonclinical research is used to evaluate a drug delivery system's or medical device's safety. Confusion has resulted from the absence of standardization in standards and recommendations about the testing and evaluation methodologies that make up a bioassessment that satisfies regulatory criteria for a medicinal device combination product (53).

## 6 Combination product regulation in India

Regulators, authorities, and outside parties should be consulted by manufacturers of combination items in India. The effectiveness or safety of the product should be proven by the manufacturer for each component. Multi-factorial research is often required for this, in which each factor is evaluated individually and in conjunction with a placebo control. Manufacturers must clearly justify every proposed active ingredient combination with a sound clinical explanation (54).

Goods like auto injectors, inhalers, pre-filled syringes, pre-filled pens for nebulizers, and transdermal patches are examples of drug-device combination goods. The following are some recommendations for creating combo items in India:

• Define the primary mode of action (PMOA)
• Research global regulatory guidelines
• Develop an appropriate CGMP quality compliance strategy
• Implement a design freeze during the combination product development process
• Seek advice from regulators, authorities, and competent third-parties regarding the documentation required to support CE mark approval applications
• Obtain the services of a Notified Body
• Demonstrate compliance with either the drug CGMPs (21 CFR parts 210 and 211) or the device Quality System (QS) regulation (21 CFR part 820) (55).

The Drugs and Cosmetics Act of 1940 and the Drugs and Cosmetics Rules of 1945 govern combination goods in India. Additionally, called Fixed Dose Combinations (FDCs), they are. Medical device regulation is handled by the Drug Controller General of India (DCGI) under the Central Drugs Standard Control Organization (CDSCO). No person or business may enter into a combination that has or is likely to have a negative impact on competition in the relevant Indian market, according to Section 6 of the Act. A combination's active ingredients ought to all function for about the same amount of time. If not, the applicant must provide an explanation and justification for the combination. As part of the Plant Master File (PMF) application procedure, the ISO 13485 certification is necessary to validate the quality system(s) of the legitimate and/or real production facilities (15).

Table 3 facilitates comprehension of the regulatory framework in India, aiding stakeholders in navigating the regulatory landscape and ensuring compliance with applicable regulations for combination products.

**Table 3:** Combination products regulations in India.

Regulation Aspect	Description
Governing Legislation	Drugs and Cosmetics Act of 1940 and Drugs and Cosmetics Rules of 1945.
Regulatory Authority	Drug Controller General of India (DCGI) under the Central Drugs Standard Control Organization (CDSCO).
Product Categorization	Combination products, also known as Fixed Dose Combinations (FDCs), which integrate drugs and medical devices.
Competition Regulation	Section 6 of the Drugs and Cosmetics Act prohibits combinations likely to have a negative impact on competition in the relevant Indian market.
Active Ingredients’ Functionality	Active ingredients in combinations should function for about the same duration; any variations require justification.
ISO 13485 Certification	ISO 13485 certification is necessary for the validation of quality systems of production facilities.
Plant Master File (PMF) Application	Part of the application procedure, providing information on the quality system(s) of legitimate production facilities.
Regulatory Approval Process	Manufacturers must prove the effectiveness and safety of each component, often requiring multi-factorial research.
Regulatory Recommendations	– Define the primary mode of action (PMOA).—Research global regulatory guidelines.—Develop a CGMP quality compliance strategy.—Implement a design freeze during development.—Seek advice from regulators and competent third-parties for CE mark approval applications.—Obtain services of a Notified Body.—Demonstrate compliance with either drug CGMPs or device Quality System regulation.

## 7 Future direction of combination products

Future directions in the field of combination products hold promise and challenges, shaped by emerging trends and technological advancements. Collaboration between regulatory bodies and industry stakeholders is likely to intensify, fostering the development of harmonized global standards to streamline the regulatory process. The integration of digital technologies, such as real-world evidence and smart devices, is poised to revolutionize efficacy assessments and post-market surveillance, providing richer and more dynamic data sets (56–58).

Regulatory changes may focus on refining classification processes and creating more tailored pathways for combination products. The need for increased flexibility to accommodate evolving technologies and interdisciplinary innovations may drive regulatory frameworks to become more adaptive and responsive (56).

Additionally, the emphasis on patient-centric healthcare may lead to enhanced patient involvement in the development and monitoring of combination products. Patient-reported outcomes and experiences could become integral components of efficacy assessments, reflecting a broader shift toward personalized and patient-centered care.

As technological advancements continue, manufacturers may explore novel combinations, such as integrating advanced biomaterials or incorporating artificial intelligence into combination products. This could usher in a new era of smart and responsive healthcare solutions.

## 8 Significant importance in the field of pharmaceuticals and natural products for several reasons

### 8.1 Innovation in healthcare

Combination products represent a frontier of innovation in healthcare, offering novel therapeutic modalities that can address unmet medical needs. They have the potential to revolutionize treatment outcomes and patient care by combining drugs, devices, and biologics in synergistic ways (58).

### 8.2 Complex regulatory landscape

The intersection of drugs, devices, and biologics in combination products creates a complex regulatory environment. Understanding and navigating this landscape is crucial for manufacturers, regulators, and healthcare professionals to ensure that these products meet rigorous safety and efficacy standards (59).

### 8.3 Patient safety

The safety of patients is paramount in healthcare. Combination products introduce unique challenges due to their diverse components, potential interactions, and the need for comprehensive risk assessment and mitigation strategies. Ensuring safety is critical for building trust among patients and healthcare providers (60).

### 8.4 Efficacy and therapeutic benefits

Evaluating the efficacy of combination products is essential for demonstrating their clinical utility and therapeutic benefits. Robust efficacy assessments, including clinical trials and post-market surveillance, are necessary to demonstrate that these products deliver on their intended outcome (56).

### 8.5 Regulatory harmonization and collaboration

Collaboration between regulatory agencies, industry stakeholders, and healthcare professionals is essential for harmonizing regulations, sharing best practices, and fostering innovation in the development and assessment of combination products. This collaboration can help streamline regulatory processes and ensure consistent standards across different jurisdictions (2, 61).

### 8.6 Future directions and technological advancements

The future of combination products holds promise for continued innovation and technological advancements. As new technologies emerge, such as digital health solutions and advanced biomaterials, the regulatory framework must adapt to accommodate these innovations while maintaining rigorous standards for safety and efficacy (62).

## 9 Conclusion

The dynamic landscape of combination products presents a complex interplay of regulatory challenges, safety considerations, and efficacy assessments. Manufacturers must navigate the intricate classification processes and collaborate seamlessly with regulatory bodies, particularly the FDA, to ensure a streamlined and comprehensive approach. Safety considerations demand meticulous risk assessment and mitigation strategies to address potential complications arising from the amalgamation of drugs, devices, and biologics. the development and regulation of combination products stand at the forefront of innovation in healthcare, offering immense potential to improve patient outcomes and address complex medical challenges. However, realizing this potential requires a concerted effort from manufacturers, regulators, healthcare professionals, and patients alike.

Navigating the intricate regulatory landscape, addressing safety considerations, and evaluating efficacy are paramount to ensuring the success of combination products. Collaboration between regulatory agencies, interdisciplinary expertise, and adherence to evolving guidelines are essential for meeting rigorous standards and fostering trust in these innovative therapies.

As we look towards the future, continued collaboration, regulatory harmonization, and technological advancements will be key drivers of progress in the field of combination products. By prioritizing patient safety, embracing innovation, and maintaining a commitment to excellence, we can unlock the full potential of these transformative medical interventions, ultimately improving the lives of patients around the world.

## References

[1] Burns LR Lawrence D Sammut S. Chapter 8. Healthcare innovation across sectors: convergences and divergences. In: Burns LR, editor. The Business of Healthcare Innovation. Philadelphia, PA: Penn Libraries, University of Pennsylvania (2012). p. 515–63.

[2] Tsourounis M Stuart J Smith M Toscani M Barone J. Challenges in the development of drug/device and biologic/device combination products in the United States and European union: a summary from the 2013 DIA meeting on combination products. Ther Innov Regul Sci. (2015) 49(2):239–48. 10.1177/2168479014553900 30222416

[3] Sun G Zhou Y-H. AI In healthcare: navigating opportunities and challenges in digital communication. Front Digit Health. (2023) 5:1–5. 1291132.10.3389/fdgth.2023.1291132 10.3389/fdgth.2023.1291132 PMC10763230 38173911

[4] Sachdeva P Kaur K Fatima S Mahak FNU Noman M Siddenthi SM Advancements in myocardial infarction management: exploring novel approaches and strategies. Cureus. (2023) 15:9. 10.7759/cureus.18617 PMC10587445 37868550

[5] Avula MN Grainger DW. Addressing medical device challenges with drug–device combinations. Drug-Device Combinations Chronic Dis. (2015) 11(1):1–38. 10.1002/9781119002956.ch01

[6] Sen S Chakraborty R. Toward the integration and advancement of herbal medicine: a focus on traditional Indian medicine. Bot Targets Ther. (2015) 2(1):33–44. 10.2147/BTAT.S66308

[7] Kazdin AE. Evidence-based treatment and practice: new opportunities to bridge clinical research and practice, enhance the knowledge base, and improve patient care. Am Psychol. (2008) 63(3):146. 10.1037/0003-066X.63.3.146 18377105

[8] Gelijns A Rosenberg N. The dynamics of technological change in medicine. Health Aff. (1994) 13(3):28–46. 10.1377/hlthaff.13.3.28 7927160

[9] Elbe S. Security and global health. Polity. (2010) 5(3):S273–4.

[10] Bhoop BS. Quality by design (QbD) for holistic pharma excellence and regulatory compliance. Pharm Times. (2014) 46(8):26–33. https://www.researchgate.net/publication/267034196

[11] Courtney B Bond KC Maher C. Regulatory underpinnings of global health security: fDA's Roles in preventing, detecting, and responding to global health threats. Biosecur Bioterror. (2014) 12(5):239–46. 10.1089/bsp.2014.0043 25254912 PMC4171126

[12] Macdonald JC Isom DC Evans DD Page KJ. Digital innovation in medicinal product regulatory submission, review, and approvals to create a dynamic regulatory ecosystem—are we ready for a revolution? Front Med (Lausanne). (2021) 8:660808. 10.3389/fmed.2021.660808 34109196 PMC8183468

[13] Oikonomou E Carthey J Macrae C Vincent C. Patient safety regulation in the NHS: mapping the regulatory landscape of healthcare. BMJ Open. (2019) 9(7):e028663. 10.1136/bmjopen-2018-028663 31289082 PMC6615819

[14] Paradise J. Reassessing safety for nanotechnology combination products: what do biosimilars add to regulatory challenges for the FDA. Louis ULJ. (2011) 56:465.

[15] Chisholm O Critchley H. Future directions in regulatory affairs. Front Med (Lausanne). (2023) 9:1082384. 10.3389/fmed.2022.1082384 36698838 PMC9868628

[16] Oberweis CV Marchal JA López-Ruiz E Gálvez-Martín P. A worldwide overview of regulatory frameworks for tissue-based products. Tissue Eng Part B. (2020) 26(2):181–96. 10.1089/ten.teb.2020.0221 31910099

[17] Aagaard TS. Regulatory overlap, overlapping legal fields, and statutory discontinuities. Va Envtl LJ. (2011) 29:237. 10.2139/ssrn.1766745

[18] Opderbeck David W. “Artificial intelligence in pharmaceuticals, biologics, and medical devices: present and future regulatory models.” Fordham L Rev*.* 88 (2019): 553.

[19] Lemmens T Gibson S. Decreasing the data deficit: improving post-market surveillance in pharmaceutical regulation. McGill Law J. (2014) 59(4):943–88. 10.1017/S0020859014000628

[20] Domingo-Lopez DA Lattanzi G Schreiber LH Wallace EJ Wylie R O’Sullivan J Medical devices, smart drug delivery, wearables and technology for the treatment of diabetes Mellitus. Adv Drug Delivery Rev. (2022) 185:114280. 10.1016/j.addr.2022.114280 35405298

[21] Nam K Henderson NC Rohan P Woo EJ Russek-Cohen E. Logistic regression likelihood ratio test analysis for detecting signals of adverse events in post-market safety surveillance. J Biopharm Stat. (2017) 27(6):990–1008. 10.1080/10543406.2017.1332039 28346083

[22] Rägo L Santoso B. Drug regulation: history, present and future. Drug Benefits Risks. (2008) 2:65–77.

[23] Dobesh PP Stacy ZA Ansara AJ Enders JM. Drug-eluting stents: a mechanical and pharmacologic approach to coronary artery disease. Pharmacotherapy J Human Pharmacol Drug Therapy. (2004) 24(11):1554–77. 10.1592/phco.24.17.1554.38048 15537561

[24] Guttieres D Sinskey AJ Springs SL. Modeling framework to evaluate vaccine strategies against the COVID-19 pandemic. Systems. (2021) 9(1):4. 10.3390/systems9010004

[25] Leenes R Palmerini E Koops B-J Bertolini A Salvini P Lucivero F. Regulatory challenges of robotics: some guidelines for addressing legal and ethical issues. Law Innov Technol. (2017) 9(1):1–44. 10.1080/17579961.2017.1337146

[26] Kayode-Ajala O. Establishing cyber resilience in developing countries: an exploratory investigation into institutional, legal, financial, and social challenges. Int J Sustainable Infrastruct Cities Soc. (2023) 8(9):1–10. 10.1080/23789689.2022.2067259

[27] Liras A. Future research and therapeutic applications of human stem cells: general, regulatory, and bioethical aspects. J Transl Med. (2010) 8:1–15. 10.1186/1479-5876-8-131 21143967 PMC3014893

[28] Chan S Medina Arellano M. Genome editing and international regulatory challenges: lessons from Mexico. Ethics Med Public Health. (2016) 2(3):426–34. 10.1016/j.jemep.2016.05.007

[29] Sugarman J Bredenoord AL. Real-time ethics engagement in biomedical research: ethics from bench to bedside. EMBO Rep. (2020) 21(2):e49919. 10.15252/embr.201949919 31944538 PMC7001493

[30] Moradi S Mahdizadeh H Šarić T Kim J Harati J Shahsavarani H Research and therapy with induced pluripotent stem cells (iPSCs): social, legal, and ethical considerations. Stem Cell Res Ther. (2019) 10(1):1–13. 10.1186/s13287-019-1377-3 31753034 PMC6873767

[31] Schiza EC Kyprianou TC Petkov N Schizas CN. Proposal for an ehealth based ecosystem serving national healthcare. IEEE J Biomed Health Inform. (2018) 23(3):1346–57. 10.1109/JBHI.2018.2801420 29993757

[32] Domingues C Jarak I Veiga F Dourado M Figueiras A. Pediatric drug development: reviewing challenges and opportunities by tracking innovative therapies. Pharmaceutics. (2023) 15(10):2431. 10.3390/pharmaceutics15102431 37896191 PMC10610377

[33] Valverde JL Weissenberg P. The Challenges of the New EU Pharmaceutical Legislation. 6. IOS Press (2005).

[34] European MedicinesAgency. Medical Devices—European Medicines Agency. Amsterdam: European Medicines Agency (2023). Available online at: https://www.ema.europa.eu/en/humanregulatory/overview/medical-devices (Accessed November 23, 2023)

[35] Sardella M Belcher G Lungu C Ignoni T Camisa M Stenver DI Monitoring the manufacturing and quality of medicines: a fundamental task of pharmacovigilance. Ther Adv Drug Saf. (2021) 12:1–17. 10.1177/20420986211038436 PMC8361554 34394910

[36] Hirani K Bansinath M Mittal R Lemos JR Adis E Poojari P Eyes on the prize: decoding the ophthalmic product regulations and intricacies of the US food and drug administration approval. J Ocul Pharmacol Ther. (2023) 39(8):572–82. 10.1089/jop.2022.0238 37797226

[37] Darrow JJ Avorn J Kesselheim AS. FDA approval and regulation of pharmaceuticals, 1983–2018. Jama. (2020) 323(2):164–76. 10.1001/jama.2019.16980 31935033

[38] Corbin J Walker AJ. FDA Overview. In: Translational Radiation Oncology. Boston: Academic Press (2023). p. 453–7. 10.1016/B978-0-323-88423-5.00019-4

[39] U.S. Food and Drug Administration. Classify Your Medical Device. Available online at: https://www.fda.gov/medical-devices/overview-de regulation/classify-your-medical-device (February 7, 2020).

[40] Valencia G Oscar A Suppadungsuk S Thongprayoon C Miao J Tangpanithandee S Ethical implications of chatbot utilization in nephrology. J Pers Med. (2023) 13(9):1363. 10.3390/jpm13091150 37763131 PMC10532744

[41] Reis ME Bettencourt A Ribeiro HM. The regulatory challenges of innovative customized combination products. Front Med (Lausanne). (2022) 9:2148. 10.3389/fmed.2022.854225 PMC9354569 35935795

[42] Sharma A Luthra G. A comprehensive review of risk management in the medical device industry. J Pharm Res Int. (2023) 35(6):14–23. 10.9734/jpri/2023/v35i630325

[43] Berry D. EBOOK: Risk, Communication and Health Psychology. Berkshire: Open University Press (2004).

[44] Kohn LT Corrigan JM Donaldson MS. Setting performance standards and expectations for patient safety. In: In to Err is Human: Building a Safer Health System. Washington, DC: National Academies Press (US) (2000). 25077248

[45] https://www.accessdata.fda.gov/scripts/cdrh/cfdocs/cfcfr/cfrsearch.cfm?fr=820.3#:~:text=(b)%20Complaint%20means%20any%20written,it%20is%20released%20for%20distribution.

[46] Koban MU. Impact of health technology assessment (reimbursement) on considerations for international regulatory strategies wissenschaftliche prüfungsarbeit.

[47] Wiklund I. Assessment of patient-reported outcomes in clinical trials: the example of health-related quality of life. Fundam Clin Pharmacol. (2004) 18(3):351–63. 10.1111/j.1472-8206.2004.00265.x 15147288

[48] Zou KH Salem LA Ray A. Real-World Evidence in a Patient-Centric Digital Era. Oxon: CRC Press, Taylor and Francis Group (2022).

[49] World Health Organization. Guidance for Post-Market Surveillance and Market Surveillance of Medical Devices, Including in Vitro Diagnostics. Geneva: World Health Organization (2020).

[50] Latoz C Larkin L Huynh-Ba K. Stability considerations for drug-device combination products-21 CFR part 4 update. AAPS Open. (2023) 9(1):10. 10.1208/s12248-022-00703-8

[51] Gelijns AC. Comparing the development of drugs, devices, and clinical procedures. In: Gelijns AC, editor. Modern Methods of Clinical Investigation: Medical Innovation at the Crossroads: Volume I. Washington, DC: National Academies Press (US) (1990). 25144087

[52] Faulkner A. Medical Technology into Healthcare and Society: A Sociology of Devices, Innovation and Governance. London: Palgrave Macmillan (2008).

[53] Jayasheel BG. Regulatory requirements for marketing fixed dose combinations. Perspect Clin Res. (2010) 1(4):120. 10.4103/2229-3485.71768 21350725 PMC3043362

[54] Lewis A. Drug-device Combination Products: Delivery Technologies and Applications. Boca Raton, Boston, New York, Washington, DC: CRC Press (2009).

[55] Kondal A Murali Krishna GV Bansal D. Clinical trial regulations in India: progress and challenges arising from recent amendments to schedule Y of the drugs and cosmetics (D&C) act 1940 (D&C rules 1945). Pharmaceut Med. (2016) 30:1–13. 10.1007/s40290-015-0127-1

[56] Reis ME Bettencourt A Ribeiro HM. The regulatory challenges of innovative customized combination products. Front Med (Lausanne). (2022) 9:821094. 10.3389/fmed.2022.821094 35935795 PMC9354569

[57] Algorri M Abernathy MJ Cauchon NS Christian TR Lamm CF Moore CM. Re-envisioning pharmaceutical manufacturing: increasing agility for global patient access. J Pharm Sci. (2022) 111(3):593–607. 10.1016/j.xphs.2021.08.032 34478754

[58] Tian J Song X Wang Y Cheng M Lu S Xu W Regulatory perspectives of combination products. Bioact Mater. (2022) 10:492–503. 10.1016/j.bioactmat.2021.12.005 34901562 PMC8637005

[59] Mohiuddin AK. Framework for patient safety. Inov Pharm. (2019) 10(1). 10.24926/iip.v10i1.1637 PMC7643696 34007524

[60] DeGrazio F Paskiet D. Injectable combination product development: facilitating risk-based assessments for efficiency and patient centric outcomes. J Pharm Sci. (2020) 109(7):2101–15. 10.1016/j.xphs.2020.01.029 32272133

[61] Ofori-Asenso R Hallgreen CE De Bruin ML. Improving interactions between health technology assessment bodies and regulatory agencies: a systematic review and cross-sectional survey on processes, progress, outcomes, and challenges. Front Med (Lausanne). (2020) 7:582634. 10.3389/fmed.2020.582634 33178721 PMC7596325

[62] Reis ME Bettencourt A Ribeiro HM. The regulatory challenges of innovative customized combination products. Front Med (Lausanne). (2022) 9:821094–00. 10.3389/fmed.2022.821094 35935795 PMC9354569

